# Rehabilitation for People with Inflammatory Arthritis: Meeting the Challenges of a Changing Healthcare Landscape

**DOI:** 10.3390/jcm14165677

**Published:** 2025-08-11

**Authors:** Rikke Helene Moe, Thea P. M. Vliet Vlieland

**Affiliations:** 1 Center for Treatment of Rheumatic and Musculoskeletal Diseases (REMEDY), Diakonhjemmet Hospital, Vinderen, P.O. Box 23, No-0319 Oslo, Norway; 2 Department of Orthopaedics, Rehabilitation and Physical Therapy, Leiden University Medical Center, P.O. Box 9600, 2300 RC Leiden, The Netherlands; t.p.m.vliet_vlieland@lumc.nl; 3 Basalt Rehabilitation, Vrederustlaan 180, 2543 SW The Hague, The Netherlands; 4 Department of Physical Therapy, Faculty of Health, Leiden University of Applied Sciences, Zernikedreef 11, 2333 CK Leiden, The Netherlands

## 1. Introduction

Rheumatic and musculoskeletal diseases (RMDs) represent the leading cause of physical disability in Europe, accounting for approximately half of all Years Lived with Disabilities (YLDs) [1,2,3,4]. Among these, inflammatory RMDs such as rheumatoid arthritis (RA) and axial spondyloarthritis (axSpA) significantly contribute to the disease burden [5]. Multidisciplinary team-based rehabilitation, traditionally delivered in hospital settings, was for a long time the standard of care. However, systematic reviews reveal only limited high-quality evidence to support its efficacy, and the number of facilities offering such services is declining. Thus, advances in rethinking current rehabilitation models are needed, including emerging approaches like extended scope practice roles, shared care frameworks, e-health, and work rehabilitation interventions, all aiming to deliver flexible, individualized, and accessible services meeting the demands of people with disabilities today [6,7].

From a policy and practice perspective, key priorities include the early detection of disability, patient engagement, supporting rehabilitation within integrated, transmural care pathways, and health system reforms to address financial barriers and shortages of health professionals. The Rehabilitation 2030 initiative of the World Health Organization (WHO) and its intervention packages provide a strategic direction for strengthening rehabilitation services globally [8,9]. These intentions may, however, not manifest as visibly as anticipated due to unforeseen disruptions such as global pandemics, armed conflicts, or macroeconomic instability. We have learned that such unforeseen events can significantly influence health system stability, shift policy and resource priorities, and impede access to the appropriate services and qualified healthcare professionals for those with chronic diseases, thereby constraining the implementation and sustainability of planned interventions like rehabilitation [10].

Nevertheless, the field of rehabilitation in RMDs is continuously evolving. In 2023, we edited a Special Issue for the *Journal of Clinical Medicine* that was dedicated to physiotherapy and rehabilitation for persons living with inflammatory arthritis (IA) [11] that focused on the substantial proportion of patients living with inflammatory joint disease (IJD) who experience considerable disease impact and biopsychosocial challenges. The condition of a substantial proportion of those people could be classified as difficult-to-treat (D2T) arthritis characterized by persistent disease activity with resulting functional limitations and reduced quality of life [12,13,14,15]. The Special Issue underscored the essential role of physiotherapy and rehabilitation as a crucial element of the comprehensive disease management for this group. This paper discusses the developing landscape based on the insights presented in the Special Issue and findings from the recent literature, detected by pragmatic searches for recently published umbrella or systematic reviews or clinical trials on physiotherapy and rehabilitation in RMDs (search up to April 2025).

## 2. Results

The Special Issue, “Physiotherapy and Rehabilitation for People with Rheumatic and Musculoskeletal Diseases”, comprised seven papers representing a wide variety of themes, stretching from the assessment of functional limitations and cardiorespiratory fitness to the impact of vocational rehabilitation and promotion of physical activity, the role of pain, and the development of a new physical therapist-driven interdisciplinary rehabilitation model of care. We see these publications as a reflection of the multifaceted needs and complexity in this field of research, which are also included in recommendations for modern RMD care [16,17].

### 2.1. Assessment of Functional Disability and Cardiorespiratory Fitness

To enhance the understanding and support of personalized care in patients with severe RA, Teuwen et al. examined functional limitations, as measured by the Health Assessment Questionnaire Disability Index (HAQ-DI), in relation to a broad range of factors in a cohort of 215 Dutch patients [18]. The results indicated that higher disability scores were not associated with disease activity but closely linked to factors such as higher age, longer disease duration, unemployment, joint replacements, impaired daily functioning, and reduced physical quality of life. These findings highlight the need for a comprehensive, multidimensional assessment, in addition to evaluation of disease activity, to inform tailored care strategies in this patient population [18]. A randomized controlled trial (RCT) originating from the same Dutch research group on a longstanding, personalized exercise intervention overseen by skilled physiotherapists was shown to be more effective in improving function than usual care in people with RA and severe functional limitations, at acceptable costs from the societal perspective [19,20].

Proper specific exercise testing is another necessity to individually tailor exercise dosage in rehabilitation, a theme that was addressed in the Special Issue by a Norwegian research group. Given that cardiorespiratory fitness (CRF) is a key indicator of overall health, but laboratory-based assessments are not widely accessible, Nordén et al. evaluated the effectiveness of estimated CRF (eCRF) models in patients with IJD [21]. Their findings indicated that the eCRF models were able to detect improvements of ≥3.5 mL/kg/min in VO_2_ peak. Although the mean differences between changes in eCRF and lab-based VO_2_ peak were small, they did not fall within the predefined equivalence margins. They concluded that eCRF assessments are promising, low-cost, and accessible alternatives that may aid in identifying patients with RMDs who should be prioritized for comprehensive laboratory-based CRF testing.

### 2.2. Impact of Rehabilitation Interventions

Skinnes et al. explored the impact of rehabilitation on work ability in a large cohort of nearly 3000 patients at working age [22]. The study found significant improvements in work ability from the start of rehabilitation to the 12-month follow-up. Higher work ability at 12 months was associated with better baseline health status, whereas lower work ability was linked to cohabitation with a partner, having a greater number of diagnoses, and moderate levels of pain. A recent systematic review indicated small beneficial effects of multidisciplinary interventions on work participation [23]. Promising rehabilitation elements were exercise, vocational counselling and psychoeducational interventions, cognitive behavioral therapies, and psychosocial support. Another systematic review on vocational and work-related interventions informing EULAR lifestyle recommendations for people with RMDs included 23 primary studies and two systematic reviews [24]. It was found that many studies indicated that work participation was not likely to be detrimental and, in some cases, beneficial for RMD-specific outcomes and should therefore receive attention during consultations with health professionals. Butink et al. summarized evidence from 64 primary studies on non-pharmacological interventions to promote work participation in people with RMDs [25]. Qualitative synthesis suggested small beneficial effects, such as an effect of 11% of interventions aiming to reduce sick leave and of 6% of interventions focusing on work status and presenteeism. Effectiveness seemed to depend on contextual factors such as the nature of the disease, population risk status, intervention characteristics, and outcomes of interest, not only highlighting the importance of individual tailoring of interventions but also of more standardization of the design and reporting of vocational rehabilitation studies. Furthermore, a recent, qualitative study found that health professionals want to support people with IA to maintain their jobs but find this difficult if the patient has not informed the employer about the disease [26]. That study emphasized the need for vocational rehabilitation to support people with IA to stay in work from the time of diagnosis through treatment, municipal rehabilitation, and job clarification. A new multi-modal vocational rehabilitation outpatient intervention led by an occupational therapist is currently being evaluated in an RCT in Denmark [27]. The intervention includes an individual assessment and goal-setting session, followed by cooperation and coordination support involving all relevant partners and by help with navigation across primary and secondary care, and four group sessions with peers and individual sessions with other health professionals, dependent on identified needs. A similar intervention, yet led by a physical therapist, was developed and is currently being evaluated in the Netherlands [28].

Pester et al. reviewed the current literature on perioperative interventions aimed at facilitating physical activity to improve pain and functional outcomes related to spine surgery [29]. While it was concluded that the evidence remains limited, it appeared that post-surgical interventions offer more substantial long-term benefits, particularly in reducing disability, as compared to regular care. Integrated approaches that combine exercise with psychosocial components may further enhance outcomes, in particular fear-avoidance behavior. The authors recommend the development of brief, accessible programs and emphasize the need for future studies to incorporate both subjective and objective measures of physical activity, as well as include short- and long-term outcome assessments. Most likely, selecting patients who will benefit the most will pose a challenge in order for such programs to be (cost-) effective. Research on the importance of physical activity for people with RMDs has grown rapidly over the past couple of years, with several reviews in Sys-temic Lupus Erythematosus (SLE) published since the Special Issue in 2023 as an example. E.g., an international task force selected 15 specific recommendations regarding physical activity for persons with SLE, generally emphasizing its safety and benefits, while noting that individual medical evaluations may be necessary to exclude contraindications [30]. Aerobic and resistance training programs were encouraged, with gradual progression tailored to the individual and ideally supervised by qualified professionals. Moreover, the application of sunscreen was recommended for outdoor activities [30]. A systematic review informing the 2023 EULAR recommendations for the management of fatigue in people with RMDs identified physical activity and exercise as beneficial in reducing fatigue in individuals with SLE (SMD = −0.54, 95% CI = −1.07 to −0.01), along with other inflammatory rheumatic diseases [31]. Likewise, other recent recommendations highlight the importance of education and support for exercise, photoprotection, and psychosocial care in SLE management [32]. These recommendations further promote holistic and personalized care while identifying the need for improved evidence, education, and clinician–patient communication.

### 2.3. Pain Management and Health Care Provision

Pain is a frequent symptom of IJD, and the special issue also included results from a qualitative study by Brodin et al. that explored pain management through interviews with a sample of patients with psoriatic arthritis (*N* = 11) [33]. In the context of individually tailored treatment for this patient group, the interviews revealed an overarching theme of actively managing life while living with persistent pain. This was reflected in the processes of addressing personal vulnerability, achieving acceptance and active engagement, and focusing on meaningful change. Health professionals in rheumatology (HPR) may need to support patients in fulfilling basic psychological needs like competence, autonomy, and relatedness. Their findings underscore the importance of enhancing professional skills not only in delivering such support but also in facilitating the involvement of family members and peers in the management process.

It is noteworthy that the promotion of active pain coping strategies within osteoarthritis (OA) care models may, paradoxically, be counterproductive when it comes to health care utilization. In a longitudinal study included in the special issue, spanning ten years, Scherpenseel et al. observed that patients with hip and knee OA who employed active coping styles demonstrated higher levels of healthcare utilization compared to those adopting more passive strategies [34]. Given that active coping has long been a central component in the management of RMDs, this finding raises questions about whether such emphasis may have inadvertently diminished attention to the appropriate tailoring of effective self-management approaches. It must be noted, however, that this study did not evaluate the results of coping style on health outcomes like symptoms and function. Nevertheless, these insights are relevant for researchers and clinicians who focus on self-management and coping styles. The findings warrant further investigation and should be carefully considered in the development and evaluation of future models of care that prioritize active coping as a therapeutic objective.

### 2.4. Innovative Rehabilitation Care Models

Early detection of IJD, particularly in suspected axSpA, remains a significant clinical challenge, with diagnostic delays often spanning over several years. These delays negatively impact treatment outcomes and impede efforts to prevent comorbidities and the associated biopsychosocial burden [35]. In order to address these unfavorable consequences of the disease, Knak et al. developed an interdisciplinary outpatient rehabilitation model, coordinated by physical therapists and supported digitally by specialists in hospital-based rheumatology care [36]. Their model aimed to improve early intervention and multidisciplinary management in patients with suspected axSpA and incorporated evidence-based rehabilitation components with proven beneficial effects. The intervention did not include e-health components for the patients but another form of remote care, i.e., a telephone hotline for health professionals to discuss early detection and the selection and dosage of rehabilitation interventions among them.

Overall, remote care, in particular e-health, has become a natural and essential component of rheumatology care over the past years, offering virtual visits and remote monitoring tools that expand access to specialized care, with particular advantages for patients in rural areas. These technologies can help enhance convenience and allow for real-time data collection to inform treatment adjustments and possibly also help time physical consultations, seamlessly integrating e-health components into clinical workflows. A recently published umbrella review showed increasing evidence for the use of innovative strategies that, although mostly with low-quality evidence, proved to provide more accessible health care for people with RMDs, as compared to usual care. The most frequent outcome measures used in the studies included in that review were patient-reported outcomes [37].

## 3. Conclusions

Recent advancements in modern rheumatological rehabilitation underscore a growing commitment to appropriate assessment, the development, evaluation, and implementation of biopsychosocial models of personalized care and multidisciplinary collaboration and integration of remote care, in particular e-health, to enhance patient outcomes. The themed Special Issue of *JCM* added important knowledge to the field of rehabilitation by providing new insights into the assessment of functional limitations and disability and cardiorespiratory fitness, the impact of rehabilitation interventions, a deeper understanding of the psychosocial aspects of pain management and coping, and new models of rehabilitative care. It yielded knowledge on particular functional problems people with RMDs are facing and on feasible alternatives to the extensive time- and equipment-consuming performance-based cardiorespiratory testing by suggesting areas for the use of more feasible estimated cardiorespiratory models (e-CRF models). One of the included studies found that there is a need for more research on effective interventions to support people with RMDs to maintain work and physical activity, yet the selection of patients who will benefit most and the tailoring of interventions to individual needs remain challenging. The latter finding was also supported by a qualitative study on pain management. The continuous need for studies evaluating the effectiveness of rehabilitation interventions was underpinned by a study suggesting that an active coping strategy, which is commonly pursued in rehabilitative care, may lead to increased health care usage. Finally, a study on an interdisciplinary outpatient rehabilitation model demonstrated the potential of innovative rehabilitative strategies, incorporating, amongst others, remote care options. All of these results are important for clinicians, health care services, and health care politicians and researchers in the development of future rehabilitation models.

To address future challenges in managing RMDs, several more general key trends also merit prioritization to develop and implement effective rehabilitative strategies for prevention, secondary prevention, and treatment. First, there is a pressing need to shift focus towards prevention and the promotion of healthy lifestyles for people at risk for developing RMDs, preferably starting with education for all children in schools. Second, greater emphasis must be placed on injury prevention; for example, evidence suggests that preventing knee injuries may account for as much as 12% of knee OA prevalence [38], injuries that could be prevented by 50% with the introduction of neuromuscular training in sports [39]. Third, the field should leverage the potential of real-time, dynamically updated implementation strategies for simple, evidence-based recommendations for relatively easily available management strategies aimed at equipping the general population with knowledge and tools before initial contact with primary care providers regarding RMD-related symptoms. Finally, collaboration with different stakeholders like the pharmaceutical and engineering sectors should be strengthened to integrate non-pharmacological strategies and rehabilitation into broader treatment paradigms to optimize their delivery in terms of content, dosage, timing, and their combination with various other treatments.
